# Lack of human-like extracellular sortilin neuropathology in transgenic Alzheimer’s disease model mice and macaques

**DOI:** 10.1186/s13195-018-0370-2

**Published:** 2018-04-24

**Authors:** Feng-Qin Zhou, Juan Jiang, Chelsea M. Griffith, Peter R. Patrylo, Huaibin Cai, Yaping Chu, Xiao-Xin Yan

**Affiliations:** 1 Department of Anatomy and Neurobiology, Central South University School of Basic Medical Science, Changsha, 410013 Hunan China; 2 Department of Physiology, Southern Illinois University School of Medicine, Carbondale, IL 62901 USA; 3 Laboratory of Neurogenetics, National Institute on Aging, National Institutes of Health, Bethesda, MD 20892 USA; 4 Department of Neurological Sciences, Rush University Medical Center, Chicago, IL 60612 USA; 5 Department of Anatomy and Neurobiology, Central South University Xiangya School of Medicine, Changsha, Hunan China

**Keywords:** β-Amyloid, Brain aging, Dementia, Neurodegenerative diseases, Vps10p

## Abstract

**Background:**

Alzheimer’s disease (AD) is a devastating neurodegenerative disorder bearing multiple pathological hallmarks suggestive of complex cellular/molecular interplay during pathogenesis. Transgenic mice and nonhuman primates are used as disease models for mechanistic and translational research into AD; the extent to which these animal models recapitulate AD-type neuropathology is an issue of importance. Putative C-terminal fragments from sortilin, a member of the vacuolar protein sorting 10 protein (Vps10p) family, have recently been shown to deposit in the neuritic β-amyloid (Aβ) plaques in the human brain.

**Methods:**

We set out to explore if extracellular sortilin neuropathology exists in AD-related transgenic mice and nonhuman primates. Brains from different transgenic strains and ages developed overt cerebral Aβ deposition, including the β-amyloid precursor protein and presenilin 1 double-transgenic (APP/PS1) mice at ~ 14 months of age, the five familial Alzheimer’s disease mutations transgenic (5×FAD) mice at ~ 8 months, the triple-transgenic Alzheimer’s disease (3×Tg-AD) mice at ~ 22 months, and aged monkeys (*Macaca mulatta* and *Macaca fascicularis*) were examined. Brain samples from young transgenic mice, middle-aged/aged monkeys, and AD humans were used as negative and positive pathological controls.

**Results:**

The C-terminal sortilin antibody, which labeled senile plaques in the AD human cerebral sections, did not display extracellular immunolabeling in the transgenic mouse or aged monkey brain sections with Aβ deposition. In Western blot analysis, sortilin fragments ~ 15 kDa were not detectable in transgenic mouse cortical lysates, but they occurred in control AD lysates.

**Conclusions:**

In reference to their human brain counterparts, neuritic plaques seen in transgenic AD model mouse brains represent an incomplete form of this AD pathological hallmark. The species difference in neuritic plaque constituents also indicates more complex secondary proteopathies in the human brain relative to rodents and nonhuman primates during aging and in AD.

**Electronic supplementary material:**

The online version of this article (10.1186/s13195-018-0370-2) contains supplementary material, which is available to authorized users.

## Background

Many transgenic mouse lines are produced as animal models of Alzheimer’s disease (AD), with the majority being engineered to overexpress mutant β-amyloid precursor protein (APP) and/or presenilin 1 or 2 (PS1, PS2) genes identified from patients with early-onset familial AD (FAD) [1–3]. The commonly studied mouse lines include mice overexpressing the APP_Swed_ transgenes (Tg2576) [4], APP/PS1 double-transgenic mice (2×FAD) [5], and mice with five FAD-linked APP/PS1 mutations (5×FAD) [6], all developing β-amyloid (Aβ) deposition in the brain with age. The triple-transgenic mouse model of AD (3×Tg-AD) also harbors a mutant human tau (P301L) gene associated with frontotemporal dementia [7] and develops both plaque- and tangle-like pathologies in the brain [8]. Transgenic AD models are widely used in exploratory studies and have provided insights into the biological, pathogenic, and behavioral/cognitive underpinnings of this disease [2, 3, 9, 10]. These animal models have also served as a prime system for the development and evaluation of various AD therapeutic approaches [1, 11, 12]. However, though in many cases excellent pharmacological efficacy is established in preclinical experiments with transgenic AD models, no effective medicine has been translated to patients to date, owing to repeated failure at various stages of clinical drug trails. This has led to discussions on the extent to which the transgenic models have sufficiently recapitulated the complexity of human AD pathology [13–17].

Aging is the best known risk factor for AD, with AD-type neuropathology occurring in the normal aging human brain [18–20]. To understand AD pathogenesis from brain aging and evolutionary perspectives, aged natural animals have long been examined for the presence of plaques and tangles in the brain [21–23]. Nontransgenic mice/rats, guinea pigs, and rabbits do not appear to develop remarkable cerebral amyloidosis with age, whereas many larger mammals, such as cats and dogs, bears, and nonhuman primates show this brain lesion as they age [24–45]. Neurofibrillary tangles and intraneuronal phosphorylated tau (p-tau) accumulation are also reported in the brains of some aged animals, including nonhuman primates [26, 28–30, 33, 34, 40, 41, 43, 44]. Because of their greatly shared genetic homogeneity with *Homo sapiens*, nonhuman primates are generally considered as better models for human neurological disorders relative to nonprimates [45, 46]. Notably, it has been also proposed that AD may be unique to humans [47]. It is therefore important to explore the extent of neuropathological difference between human and nonhuman primates [47–50].

During the past several years, the vacuolar protein sorting 10 protein (Vps10p) family has attracted much attention in the AD research field [51]. A growing number of studies show that genetic variations among members of this family, including sortilin-related receptor L1 (SORL1, also known as SORLA, SORLA1, or LR11) [52–58], sortilin-related Vps10p domain-containing receptor 1 (SORCS1) [59], and sortilin [60], are related to the risk of developing AD. Further understanding of the neurobiological and neuropathological roles played by this family of proteins might extend novel AD diagnostic and therapeutic options. We recently reported that putative C-terminal fragments from sortilin can deposit in aged and AD human brains as extracellular lesions morphologically appearing as senile plaques [61]. The deposition occurs fairly selectively at neuritic amyloid plaques, as visualized with an antibody (catalog number ab16640; Abcam, Cambridge, UK) targeting the intracellular C-terminal domain of sortilin, which is evolutionarily conserved among mammals. Immunoblot analysis indicates some ~ 15 kDa fragments likely being the major deposited products. Two recent studies by other groups have suggested an involvement of sortilin in amyloid and tau pathogenesis [62, 63], with the levels of full-length sortilin increased in the neocortex of aged relative to young β-amyloid precursor protein and presenilin 1 double-transgenic (APP/PS1) mice [62].

One might expect that animal models which better recapitulate AD pathological features would be more relevant in basic and translational research into this disease. Given the importance of the senile plaque pathology in understanding AD, in the present study we sought to explore whether some commonly used transgenic AD mouse models (APP/PS1, 5×FAD, and 3×Tg-AD mice) and *Macaca* monkeys recapitulate the extracellular sortilin neuropathology first identified in humans [61].

## Methods

### Overall experimental design, brain samples, and tissue processing

Pilot experiments were carried out to assess sortilin immunolabeling with the two antibodies generated against the extracellular and intracellular C-terminal domains, respectively [62]. These experiments used cryoprotected forebrain sections from transgenic mice available from earlier studies [64, 65]. The animals were perfused at representative age time points before and after the onset of cerebral amyloid pathology: APP/PS1 mice at 2, 8, and 14 months; 5×FAD mice at 1, 2, and 6 months; and 3×Tg-AD mice at 4, 6, 12, 18, and 26 months. Overall, no plaque-like extracellular sortilin immunolabeling was observed in the processed sections. Therefore, for formal correlated anatomical/biochemical study, we obtained transgenic mice at selected age points: APP/PS1 mice at 14 months (*n* = 8) (Better Biotechnology Co., Ltd., Nanjing, China); 5×FAD mice at 8 months (*n* = 4) (The Jackson Laboratory, Bar Harbor, ME, USA), and 3×Tg-AD mice at 22 months (*n* = 4) (in-house breeding, Southern Illinois University at Carbondale). The transgenic animals at the aforementioned age points have developed robust cerebral amyloid pathology as established earlier [5, 6, 8, 64, 65]. Brain samples from C57BL/6 mice (6 months; *n* = 8) were also used as negative assay controls.

Among the APP/PS1 and C57BL/6 mice, four animals were perfused transcardially under deep anesthesia (sodium pentobarbital 100 mg/kg intraperitoneally) with normal saline followed by 4% paraformaldehyde, with the brains dissected out, postfixed, and cryoprotected in 30% sucrose before sectioning at the frontal plane at 35-μm thickness in a cryostat. Other APP/PS1 (*n* = 4) and C57BL/6 mice (*n* = 4) were perfused with cold saline only, with the brains removed, snap-frozen, and stored at − 80 °C until tissue homogenization for immunoblot analysis. The 5×FAD and 3×Tg-AD mice were also perfused transcardially with cold saline first. After removal from the skull, brains were bisected along the cerebral sagittal fissure, with half brains immersed in 4% paraformaldehyde fixative for further histological processing as with the aforementioned perfusion-fixed mouse brains, whereas the other half brains were snap-frozen for Western blotting. To allow easy identification of sections from different transgenic strains after batch-processing immunohistochemistry, 5×FAD mouse hemibrain and 3×Tg-AD mouse hemibrain were sectioned (35 μm thick) along the frontal and sagittal planes, respectively. All mice were housed individually in a light-controlled (12-h/12-h on/off) and temperature-controlled (22–25 °C) vivarium with free access to food and water.

Monkey brain sections used in the experiments in the present study were available from earlier original studies (e.g., [42, 66, 67]). Selection of the tissues/cases was based on pilot immunohistochemical assessment in a set of sections from each brain. Thus, cerebral sections used in the present study were from rhesus monkeys (*Macaca mulatta*) at middle (*n* = 2, 22, and 22.5 years old) and old (*n* = 2, 27, and 30 years old) ages without cerebral amyloid pathology, as well as from four aged (30, 30.3, 31, and 34 years old) rhesus monkeys and three aged (29, 30.5, and 32 years old) cynomolgus monkeys (*Macaca fascicularis*) with cerebral amyloid lesions. In the original studies, the monkeys were housed individually on a 12-h/12-h on/off lighting schedule with free access to food and water. They were sedated with ketamine (20 mg/kg intramuscularly) and anesthetized with sodium pentobarbital (25 mg/ kg intravenously) prior to transcardiac perfusion with normal saline followed by 4% paraformaldehyde. The brains were then removed and cryoprotected in 30% sucrose in 0.1 M sodium PBS at 4 °C. Serial sections (40 μm thick) across the cerebrum were cut frontally on a sliding frozen microtome and then stored at − 20 °C in cryoprotectant until use in the present study.

Postmortem human brains were obtained through the willed body donation program at Xiangya School of Medicine [68]. After removal from the cranium, the cerebrum was bisected, with one hemisphere fresh-frozen and another hemisphere fixed by immersion in formalin followed by histological preparation. As part of standard brain banking protocol [69], each brain was assessed for AD neuropathology with Aβ and tau immunolabeling in paraffin or cryostat sections from the temporal, prefrontal, and occipital lobes of the fixed hemisphere, with the extent of pathology (if present) scored according to Braak’s staging and the National Institutes of Health recommended guideline [70–72]. For the present study, cryoprotected sections (40 μm thick) from the frontal and temporal lobes of the brains from aged cases (*n* = 4) with an antemortem history of dementia (designated as AD cases) were used for immunohistochemical analysis. Temporal cortical samples from the frozen hemispheres of the same cases were obtained for Western blot analysis. These brains were taken with postmortem delays ≤ 18 h and showed Braak’s score of neurofibrillary tangle ≥ IV, with amyloid pathology present across all cortical lobes, including the hippocampal formation.

Animal use was in accordance with the National Institutes of Health Guide for the Care and Use of Laboratory Animals and was approved by the Institutional Animal Care and Use Committee of Central South University (mice) and Rush University (monkeys). Use of postmortem human brains was approved by the Ethics Committee for Research and Education at Xiangya School of Medicine, in compliance with the Code of Ethics of the World Medical Association (Declaration of Helsinki).

### Immunohistochemistry

Transgenic mouse, monkey, and human cerebral sections were processed in parallel for correlated examination of immunolabeling. Thus, three or four forebrain sections at different planes from one mouse, one prefrontal and one temporal lobe section from one monkey, and one frontal and one temporal lobe section from one human case, were batch-processed in each experiment for a given antibody. Adjacent sets of sections were immunohistochemically processed with the following primary antibodies: rabbit anti-sortilin intracellular C-terminal domain (1:2000, catalog number ab16640, raised against recombinant amino acids 800–831 of human sortilin, catalog number ab16686, Abcam Trading Shanghai Company Ltd., Shanghai, China), goat anti-sortilin extracellular domain (diluted at 1:2000, catalog number AF3154, raised against recombinant Gly76 to Asn753 of human sortilin, catalog number 2934-ST, R&D Systems China Co. Ltd., Shanghai, China), monoclonal mouse anti-Aβ 6E10 (1:4000, catalog number 39320, Signet Laboratories Inc., Dedham, MA, USA), rabbit anti-β-secretase (anti-BACE1) [64, 65, 73], and rabbit anti-phosphorylated tau (1:4000, catalog number T6819, Sigma-Aldrich, St. Louis, MO, USA) or mouse anti-phosphorylated tau (PHF1, 1:4000; courtesy of Dr. P. Davis). Sections were first treated free-floating with 5% H_2_O_2_ in PBS for 30 min and 5% normal horse serum in PBS with 0.3% Triton X-100 for 1 h to lower nonspecific reactivity. For the sections subjected to 6E10 immunolabeling, an antigen retrieval step with formic acid treatment (1 h at room temperature) was applied prior to the aforementioned steps. Following incubation with the primary antibodies at 4 °C overnight, the sections were reacted with biotinylated pan-specific secondary antibody (horse anti-mouse, rabbit, and goat immunoglobulin G [IgG]) at 1:400 for 1 h and with avidin-biotin complex reagents (1:400; Vector Laboratories, Burlingame, CA, USA) for another 1 h. The immunoreactive product was visualized in 0.003% H_2_O_2_ and 0.05% 3,3′-diaminobenzidine, with sections mounted on microslides, dehydrated, and coverslipped for light microscopic examination.

### Immunofluorescence

Double immunofluorescence was initiated with treatment of a batch of sections from transgenic mouse, monkey, and human cortex in PBS containing 5% donkey serum for 30 min. The sections were then incubated overnight at 4 °C with (1) mouse anti-Aβ antibody 6E10 (1:4000) and the rabbit anti-sortilin antibody (ab16640, 1:1000) or (2) 6E10 (1:4000) and rabbit anti-BACE1. Sections were then incubated at room temperature for 2 h with Alexa Fluor® 488-conjugated donkey anti-mouse IgG and Alexa Fluor® 594-conjugated donkey anti-rabbit IgG (1:200, Jackson ImmunoResearch Laboratories, Inc., West Grove, PA, USA). The monkey and human brain sections were treated with 0.1% Sudan Black to block autofluorescence after immunolabeling. All sections were counterstained with bisbenzimide (Hoechst 33342, 1:50,000, catalog number B2261, Sigma-Aldrich, St. Louis, MO, USA) and mounted with antifade medium before microscopic examination.

### Western blot analysis

Frontal cortices were blocked from the frozen whole brains or hemibrains of the mice (*n* = 4/strain). Human cortical samples were blocked from the middle temporal lobes of the frozen hemispheres. Tissue samples were homogenized by sonication in Pierce T-PER extraction buffer (Thermo Fisher Scientific, Rockford, IL, USA) containing protease inhibitors (Roche, Indianapolis, IN, USA). Resulting brain lysates were centrifuged at 15,000 × *g*, with the supernatants collected and protein concentrations measured by *DC* detergent-compatible protein assay (Bio-Rad Laboratories, Hercules, CA, USA). Extracts containing 50 μg of total protein were run in 10% or 15% sodium dodecyl sulfate-PAGE gels. Separated proteins were electrotransferred onto Trans-Blot pure nitrocellulose membranes (Bio-Rad Laboratories) and then immunoblotted with rabbit anti-sortilin (1:2000), mouse anti-Aβ 6E10 (1:4000), rabbit anti-phosphorylated tau (T6819, 1:2000), and mouse anti-glyceraldehyde-3-phosphate dehydrogenase (GAPDH, 1:5000; Millipore Shanghai Trading Company Ltd., Shanghai, China) as loading controls. The membranes were further reacted with horseradish peroxidase-conjugated goat anti-rabbit or anti-mouse IgG (1:20,000; Bio-Rad Laboratories). Immunoblot signaling was visualized with the Pierce ECL-Plus Western Blotting Substrate detection kit (Thermo Fisher Scientific), followed by X-ray film exposure and image capture in a laser scanner.

### Imaging, data analysis, and figure preparation

Immunolabeled sections were examined on an Olympus BX51 microscope (CellSens Standard; Olympus Corp., Tokyo, Japan) for basic assessment of histological integrity and immunolabeling. Light microscopic images were taken using a ×20 lens objective on a Motic Olympus microscope equipped with an automated stage and imaging system (Wuhan, China), which yielded non-edge-montaged and magnification-adjustable images covering the entire area of a glass slide. Immunofluorescent color images were captured on a Nikon confocal microscope using ×20 and ×40 lens objectives, with single-channel images extracted using the EZ-C1 FreeViewer version 3.70 software (Nikon, Tokyo, Japan). Immunoblot images were densitometrically analyzed using OptiQuant software (Packard Instrument Co., Meriden, CT, USA). Optical densities over target protein bands were measured with the rectangular selection tool, exported into Excel spreadsheets, and rearranged according to groups, with relative density levels calculated against internal references. Data were then entered into Prism software spreadsheets and graphed (GraphPad Software, La Jolla, CA, USA). Statistical analyses were carried out using a nonparametric test (Kruskal-Wallis test with post hoc Dunn’s multiple comparison using Prism 4.1 software), with the level of statistical significance set at *p* < 0.05. Figures were assembled using Photoshop 7.1 (Adobe Systems, San Jose, CA, USA).

## Results

### Sortilin antibodies displayed neuronal labeling in plaque-free mouse and monkey cerebrum

**Fig. 1:**
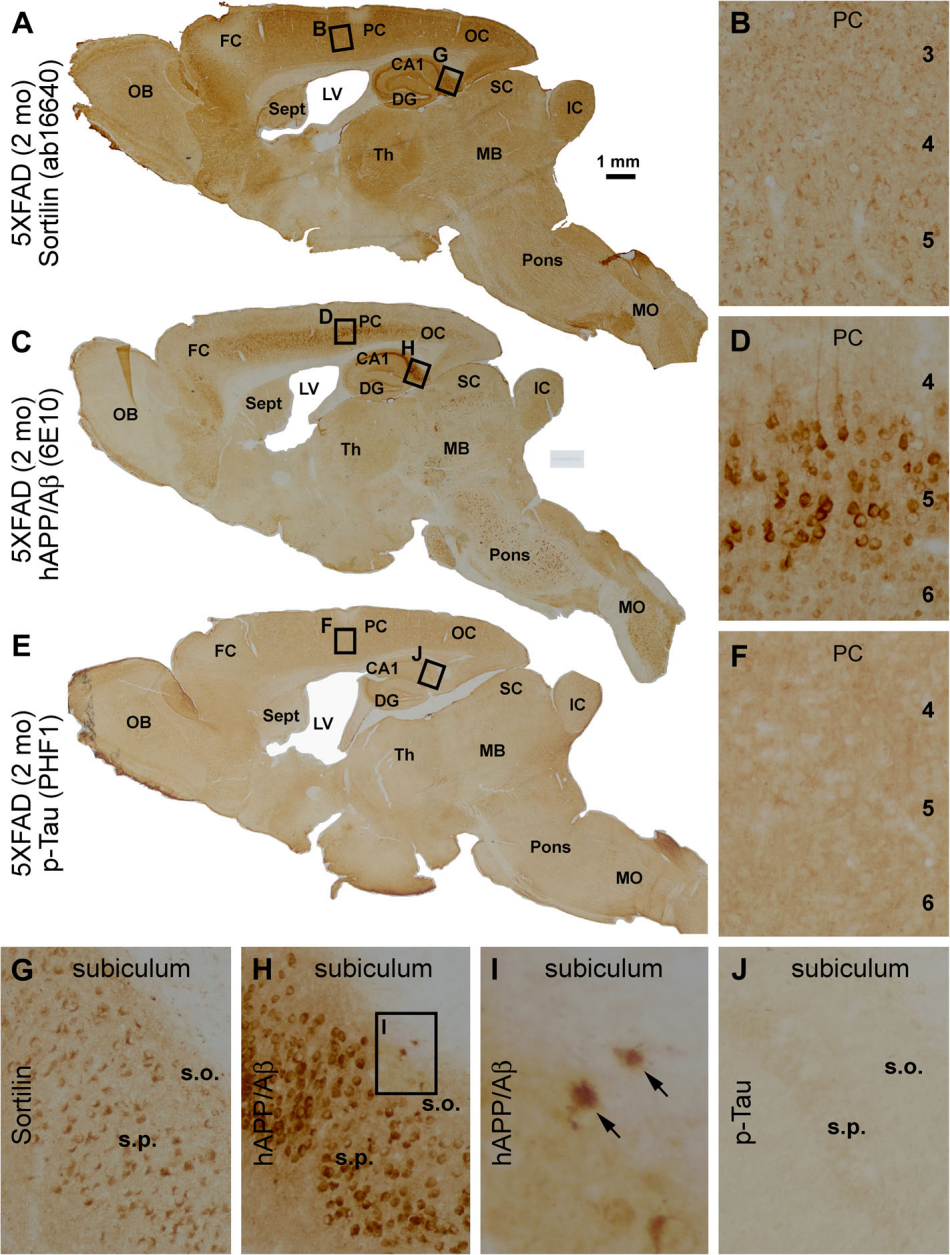
Light microscopic images showing the distribution of sortilin immunolabeling across the brain of a 2-month-old five familial Alzheimer’s disease mutations transgenic (5×FAD) mouse with transgenic background confirmation. **a**, **c**, and **e** Low-magnification images of adjacent parasagittal sections immunohistochemically stained with the rabbit sortilin antibody raised against the intracellular C-terminal domain, as well as with the monoclonal β-amyloid (Aβ) antibody 6E10 and the PHF1 mouse anti-phosphorylated tau (p-tau) antibody, for transgenic phenotype validation. Neocortical and subicular areas (*boxes*) are enlarged and shown in other panels as indicated. Sortilin immunolabeling occurs in the somata and proximal dendrites of cortical and hippocampal neurons (**a**, **b**, **g**), denser in those of relatively large somal size representing likely pyramidal neurons (**b**, **g**). The 6E10 antibody, which can recognize transgenic human β-amyloid precursor protein (hAPP) as well as Aβ, heavily labels cells in the middle portion of the cerebral cortex and stratum pyramidale (s.p.) of the hippocampus and subiculum (**b**, **d**, **h**). The heavy 6E10 immunoreactivity in layer V (**d**) and subicular (**h**) pyramidal neurons represents strong expression of transgenic hAPP in these neurons. Two extracellular miniplaques (*arrows*) are visible at the stratum oriens (s.o.) of the subiculum (**h**, **i**). The PHF1 antibody reveals background reactivity without any distinct cellular immunolabeling across the entire brain (**e**, **f**, **j**). *OB* Olfactory bulb, *FC* Frontal cortex, *PC* Parietal cortex, *OC* Occipital cortex, *Sept* Septum, *LV* Lateral ventricle, *CA1* CA1 subsector of the hippocampus, *DG* Dentate gyrus, *Th* Thalamus, *SC* Superior colliculus, *IC* Inferior colliculus, *MB* Midbrain, *MO* Medulla oblongata. Scale bar = 1 mm in (**a**) applying to (**c**, **e**), equivalent to 100 μm for (**b**–**h**, **j**) and 25 μm for (**i**)

**Fig. 2:**
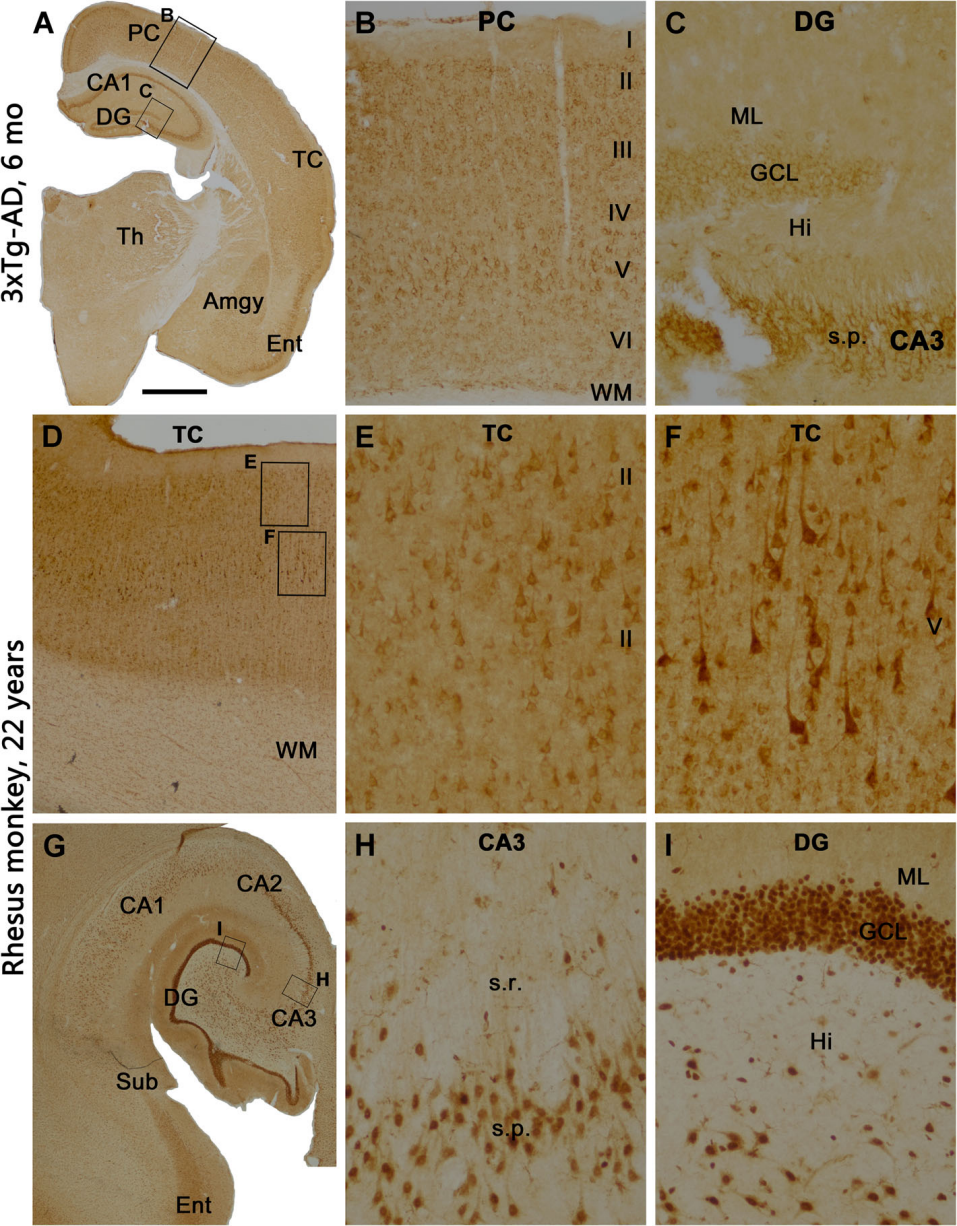
Sortilin expression pattern in the forebrain of preplaque triple-transgenic Alzheimer’s disease (3×Tg-AD) mice and middle-aged *Macaca* monkeys. The immunolabeling is visualized with the rabbit sortilin antibody. Framed areas in the left low-magnification panels (**a**, **d**, **g**) are enlarged in the middle (**b**, **e**, **h**) and right (**c**, **f**, **i**) panels. **a**–**c** Immunolabeling in a frontal brain section from a 3×Tg-AD mouse at 6 months of age. Neuronal somata and their proximal dendrites are immunolabeled, occurring over cortical layers II–VI (**b**), the amygdala (**a**), thalamic nuclei (**a**), the stratum pyramidale (s.p.) of hippocampal CA1–CA3 regions, and the granule cell layer (GCL) of the dentate gyrus (DG) (**a**, **c**). In the monkey neocortex, cellular labeling involving largely pyramidal neurons is present over the gray matter across layers II–VI (**d**–**f**), with background reactivity seen in the white matter (WM) (**d**). In the hippocampal formation (**g**), pyramidal neurons (**g**, **h**) and granule cells of the DG (**i**) are clearly labeled. The molecular layer (ML) shows neuropil labeling with intensity higher than background (**g**, **i**). Abbreviations are as in the Fig. 1 legend, as well as the following: *TC* Temporal cortex, *Ent* Entorhinal cortex, *Sub* Subiculum, *Hi* Hilus, *s.r.* Stratum radiatum. Scale bar in (**a**) = 2 mm, applying to (**g**), equivalent to 1 mm for (**d**), 500 μm for (**b**) and 200 μm for (**c**, **e**, **f**, **h**, **i**)

In a recent study that first documented the extracellular sortilin pathology in the human cerebrum, we used a goat antibody against the extracellular domain and a rabbit antibody against the intracellular C-terminal domain of sortilin [61]. For the first part of the present study, we carried out experiments using both antibodies to assess if sortilin expression in the forebrain could exhibit any impressive microscopic alteration in preplaque transgenic model mice relative to C57BL/6 control mice, as well as in amyloid-free old monkeys relative to middle-aged animals. The immunolabeling pattern revealed by the two antibodies was identical, with primarily neuronal reactivity localized to the cerebral gray matter. To avoid redundancy, we report only data derived from the labeling of the sortilin C-terminal antibody in this report, given that this antibody labels the plaque-like lesions, in addition to the cellular profiles (neuronal somata and large dendrites) that are equivalently marked by the goat antibody. Overall, in sections obtained from the three transgenic lines at all preplaque ages, the C-terminal sortilin antibody labeled the somata and dendrites of cortical and hippocampal neurons (Figs. 1 and 2). For example, in 2-month-old 5×FAD mice with miniplaques first onset at the subiculum [64], only cellular profiles were labeled with the rabbit sortilin antibody across the entire brain (Fig. 1a–d, g–i). This same cellular-only sortilin immunolabeling pattern was also noted in the brains of 2- and 8-month-old APP/PS1 mice (not shown) and 4- to 18-month-old 3×Tg-AD mice (Fig. 2a–c). Moreover, in sections from middle-aged (Fig. 2d–i) as well as amyloid-free old rhesus monkeys (not shown), sortilin immunoreactivity occurred in neuronal somata and dendrites. Therefore, the immunolabeling observed in the cerebral cortex and hippocampal formation of plaque-free transgenic mice and monkeys reflected a normal expression pattern of sortilin comparable to that seen in middle-aged humans [61].

### Extracellular sortilin deposition was not visualized in transgenic mouse and monkey brains with cerebral amyloidosis

**Fig. 3:**
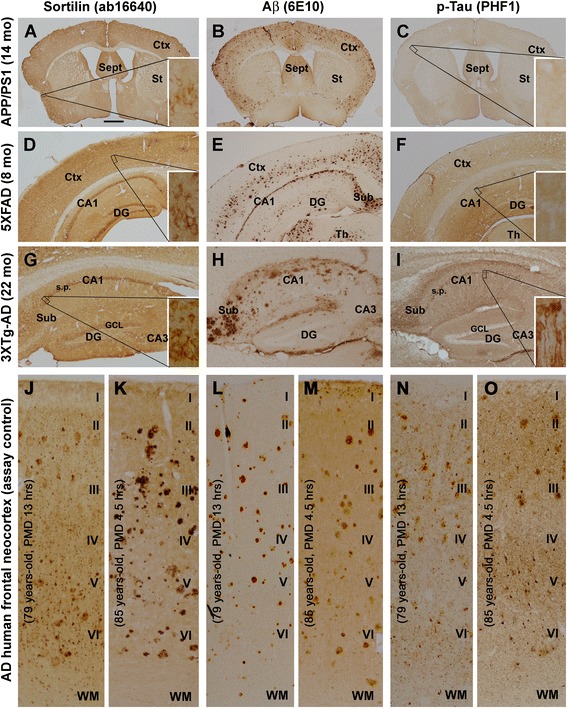
Representative light microscopic images illustrating a lack of extracellular sortilin pathology in transgenic mouse forebrain, regardless of brain region and presence of β-amyloid (Aβ) and tau pathologies. Sortilin immunolabeling visualized with the rabbit antibody is shown in the left panels from the amyloid precursor protein and presenilin 1 double-transgenic (APP/PS1), five familial Alzheimer’s disease mutations transgenic (5×FAD), and triple-transgenic Alzheimer’s disease (3×Tg-AD) mice at the indicated ages (**a**, **d**, **g**), as well as from two human cases with Alzheimer’s disease (AD) (**j**, **k**). Aβ immunolabeling in adjacent sections is visualized with the 6E10 antibody, arranged as the middle panels correspondingly (**b**, **e**, **h**, **l**, **m**). The right panels show immunoreactivity displayed with the PHF1 mouse anti-phosphorylated tau (p-tau) antibody from another set of neighboring sections from the cases (**c**, **f**, **i**, **n**, **o**). *Boxed areas* are enlarged as insets in the panels as indicated. Different from the human cortex as pathological positive control (**j**, **k**), there is no plaque-like sortilin labeling seen in the transgenic mouse sections, whereas neuronal labeling is seen in the cortex and hippocampal formation (**a**, **d**, **g**, insets). Extracellular Aβ deposition is clearly present in the transgenic mouse cortex and hippocampal formation (**b**, **e**, **h**) as well as in the human cortex (**l**, **m**). p-Tau immunolabeling is background-like in the APP/PS1 and 5×FAD sections (**c**, **f**), whereas in the 3×Tg-AD mouse section, pyramidal neurons in the subiculum and CA1 area are clearly labeled (**i**, insets). In the human cortex, p-tau immunolabeling is seen in neuronal somata and processes as well as at neuritic plaques (**n**, **o**). Abbreviations are as defined in Fig. 1 legend, as well as the following: *Ctx* Cortex, *PMD* Postmortem delay in hours, *WM* White matter. Scale bar = 1 mm in (**a**) applying to (**b**, **c**), equivalent to 500 μm for (**d**–**i**) and 250 μm for (**j**–**o**)

Sections from the three transgenic lines and aged/AD humans were stained in a batch-processing manner with the sortilin C-terminal, 6E10, and p-tau antibodies using the avidin-biotin complex immunohistochemical method for comparative analyses. The sortilin C-terminal antibody exhibited only cellular but not plaque-like labeling in sections across the entire rostrocaudal dimension of the cerebrum from the 14-month-old APP/PS1 mice (Fig. 3a, showing an image of a coronal section at the striatum level covering both hemispheres), 8-month-old 5×FAD mice (Fig. 3d, coronal section at the mid-hippocampal level covering one hemisphere), and 22-month-old 3×Tg-AD mice (Fig. 3g, sagittal cerebral section passing the middle part of hippocampus). In adjacent sections, extensive plaque-like immunoreactivity was labeled with the 6E10 antibody in the cortex, hippocampal formation, or subcortical structures in all the transgenic brains (Fig. 3b, e, h). Consistent with the transgenic background, no neuronal profiles with enhanced p-tau immunolabeling were seen in the APP/PS1 (Fig. 3c) and 5×FAD (Figs. 2e, f, j; 3f) mouse brains. In contrast, cells with strong p-tau immunoreactivity were present in the cortex and hippocampal formation of 22-month-old 3×Tg-AD mice, with the labeling distinctly visualizing the subicular and CA1 pyramidal neurons and processes (Fig. 3i, inset). In batch-processed neocortical sections from AD cases, neuronal and plaque-like profiles were labeled by the C-terminal sortilin antibody (Fig. 3j, k), with aberrant Aβ (Fig. 3l, m) and p-tau (Fig. 3n, o) immunoreactivity confirmed in neighboring sections.

**Fig. 4:**
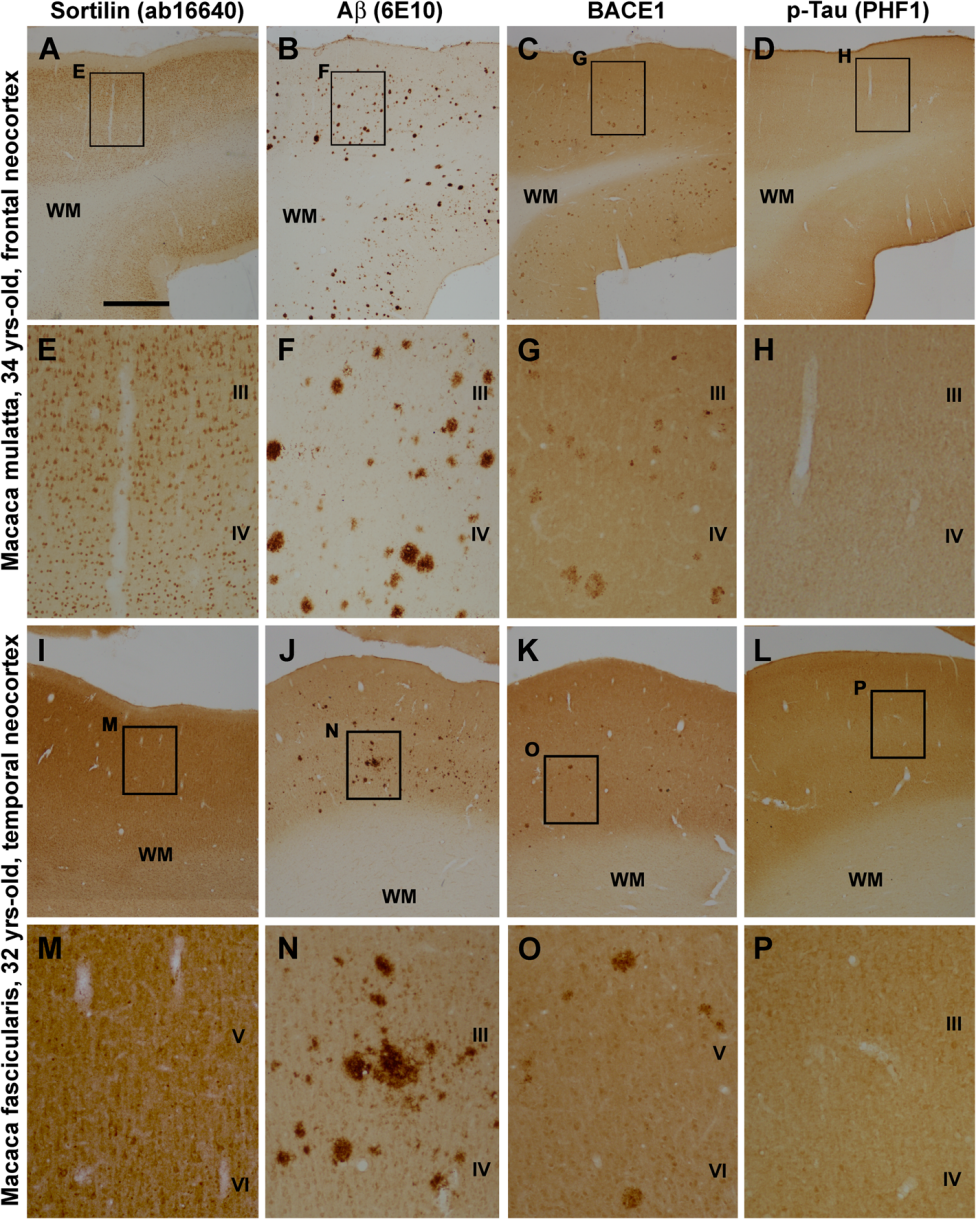
Representative light microscopic images illustrating a lack of extracellular sortilin pathology in aged monkey neocortex with overt amyloid plaque pathology. Shown are images taken from parallel processed sections from the prefrontal lobe of a 34-year-old rhesus monkey (**a**–**h**) and from the temporal lobe of a 32-year-old cynomolgus monkey (**i**–**p**), with framed areas in the low-magnification images enlarged as indicated. Sortilin immunoreactivity in the neocortex is exclusively cellular, largely present in layers II–VI with little labeling in the white matter (WM) (**a**, **e**, **i**, **m**). β-Amyloid (Aβ) plaques labeled with the 6E10 antibody are present in the middle and lower portions of the cortex in fairly large amounts (**b**, **f**, **j**, **n**). Clusters of dystrophic neurites show increased β-secretase (BACE1) immunoreactivity relative to background (**c**, **g**, **k**, **o**) and distributed over the cortex with a laminar pattern similar to that of Aβ plaques. No cellular labeling is visualized by the PHF1 p-tau antibody in the monkey neocortex. The panels shown are derived from corresponding montaged images captured on an automated Olympus Motic microscope across the entire hemispheric area, available online as Additional file 1: Figures S1–S8. Abbreviations are as defined in Fig. 1. Scale bar = 2 mm in (**a**) applying to (**b**–**d**, **i**–**l**), equivalent to 500 μm for (**e**–**h**, **m**–**p**)

Using the staining approach described above, we carried out immunolabeling with the sortilin C-terminal antibody relative to Aβ and p-tau immunolabeling in aged monkey cases identified during pilot assessment of plaque pathology, using frontal and temporal lobe sections. The sortilin immunoreactivity again occurred exclusively in cellular (neuronal) profiles in these aged rhesus (Fig. 4a, e) and cynomolgus (Fig. 4i, m) monkeys, whereas 6E10 immunoreactive plaques were clearly present in neighboring sections (Fig. 4b, f, j, n). To cross-validate the presence of neuritic plaque pathology, another set of neighboring sections were immunolabeled with BACE1 antibody. Thus, BACE1-labeled profiles arranged as neuritic clusters occurred in adjacent sections (Fig. 4c, g, k, o). The regional/laminar distribution appeared comparable between 6E10-labeled plaques and BACE1-labeled neuritic clusters either as assessed over a large cortical region (entire hemispheric area) or in reference to different monkeys (Fig. 4 and Additional file 1: Figs. S1–S3, S5–S7). In contrast to the positive labeling in human cortex (Fig. 3n, o), the two p-tau antibodies did not visualize neuronal profiles in the cerebral cortex and hippocampal formation of the aged rhesus or cynomolgus monkeys (Fig. 4d, h, l, p; Additional file 1: Figs. S4, S8).

**Fig. 5:**
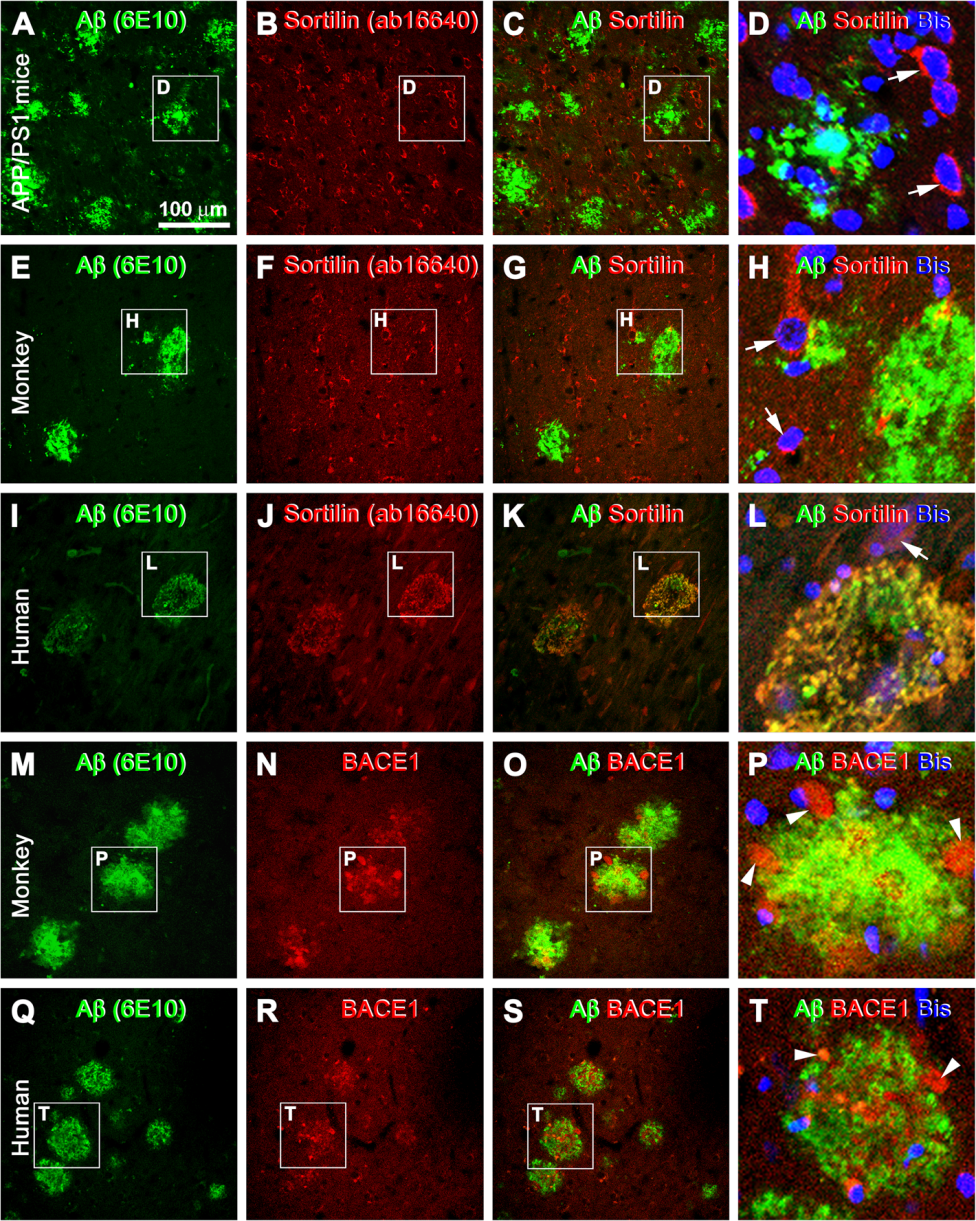
Double-immunofluorescence characterization of sortilin labeling relative to amyloid plaque pathology in transgenic mouse, aged monkey, and human neocortex. Species, antibodies, and fluorescence channels are indicated. The framed areas are enlarged as the last panel for each set of double-immunofluorescence images, with bisbenzimide (Bis) nuclear stain included (**d**, **h**, **l**, **p**, **t**). **a**–**d** Double-immunofluorescence with the rabbit anti-sortilin and 6E10 antibodies in the parietal neocortex of the amyloid precursor protein and presenilin 1 double-transgenic (APP/PS1) model (14 months of age) as an example of the lack of extracellular sortilin labeling in transgenic mouse forebrain with β-amyloid (Aβ) deposition. **e**–**h** The same situation as above in aged monkey neocortex (from a 34-year-old rhesus monkey). Note that neuronal somata and proximal dendrites (*white arrows*) show distinct sortilin immunolabeling in both the transgenic (**b**, **d**) and monkey (**f**, **h**) cortex. **i**–**l** Colocalization of sortilin and Aβ immunofluorescence in two circular plaques in human cortex (positive control). **m**–**t** Neuritic plaques in the monkey (**m**–**p**) and human (**q**–**t**) cortex consisted of extracellular Aβ deposits intermingling with dystrophic neurites (P and T, *white arrowheads*) exhibiting strong β-secretase 1 (BACE1) immunoreactivity. Scale bar = 100 μm in (**a**) applying to (**b**, **c**, **e**-**g**, **i**–**k**, **m**–**o**, **q**–**s**), equivalent to 33 μm for (**d**, **h**, **l**, **p**, **t**)

Double immunofluorescence was also used to confirm the immunohistochemical results described above. Thus, in 6E10 and sortilin double-stained transgenic mouse and aged monkey cortical sections, Aβ immunofluorescence occurred extracellularly, whereas sortilin signal was localized exclusively to neuronal somata and dendrites (Fig. 5a–h). In contrast, in batch-processed human cortex, colocalized 6E10/sortilin immunofluorescence was clearly seen at extracellular plaques (Fig. 5i–l). The neuritic nature of cortical amyloid plaques in the monkey (Fig. 5m–p) and human (Fig. 5q–t) sections was confirmed by double immunofluorescence, as indicated by the occurrence of localized extracellular Aβ labeling around clusters of BACE1 immunoreactive dystrophic neurites.

### The ~ 15 kDa sortilin fragments were not detectable in transgenic mouse cortical lysates

**Fig. 6:**
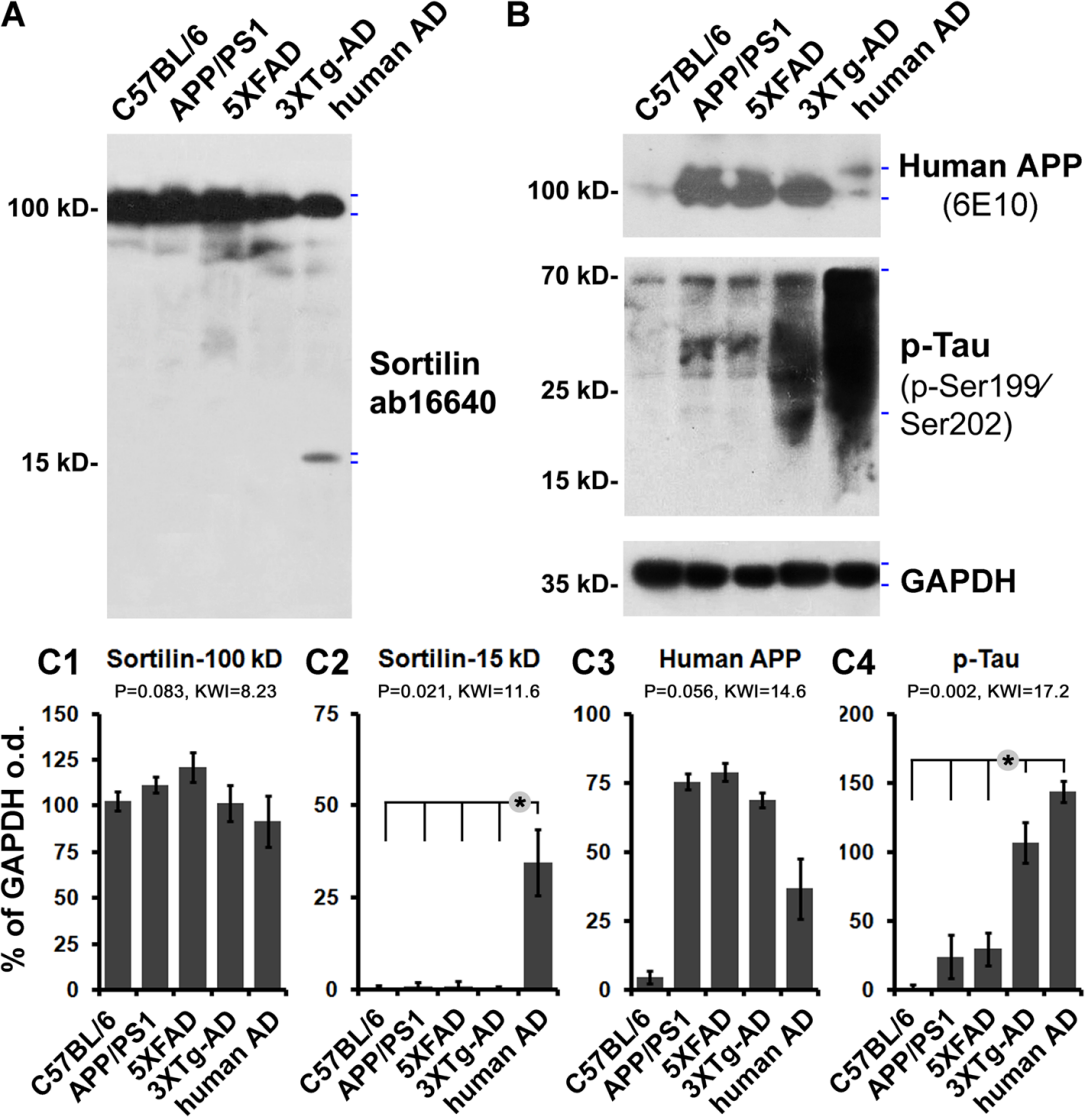
Comparative immunoblot assessment of sortilin relative to human β-amyloid precursor protein (APP) and phosphorylated tau (p-tau) protein products in transgenic mouse cortical extracts. Transgenic tissue homogenates are from the same age groups of amyloid precursor protein and presenilin 1 double-transgenic (APP/PS1), five familial Alzheimer’s disease mutations transgenic (5×FAD), and triple-transgenic Alzheimer’s disease (3×Tg-AD) mice used in histological studies, with lysates from adult C57BL/6 mice and from human patients with AD serving as controls. **a** and **b** Western blot images from one batch-processed set of samples. **c1**–**c4** Quantitative summaries of the protein levels relative to glyceraldehyde-3-phosphate dehydrogenase (GAPDH) as an internal control, expressed as a percentage of GAPDH optical density (o.d.) for the groups (*n* = 4/group). Levels of the ~ 100 kDa sortilin band representing the full-length protein are comparable between the groups (**a**, **c1**). The ~ 15 kDa sortilin band is not readily seen in all mouse brain lysates, in contrast to the human tissue as positive control (**a**, **c2**). The human APP protein bands (~ 100 kDa) detected by the 6E10 antibody are distinct in the lysates from the three transgenic models and human cortex, but not in that of the C57BL/6 control (**b**, **c3**). Immunoblotted p-tau products migrate as a smear of bands (20–70 kDa), mostly abundant in the human lysates but clearly present in the 3×Tg-AD samples, with minimal amounts in the C57BL/6, APP/PS1, and 5×FAD samples (**b**, **c4**). Hash marks to the right of the immunoblot images indicate the band(s) used for densitometry. Statistical results (Kruskal–Wallis nonparametric test with Dunn’s multiple post hoc comparison) are shown in the bar graphs, with the asterisks indicating significant intergroup differences

We concurrently analyzed cortical lysates from the three transgenic mouse models (*n* = 4) using samples from C57BL/6 mice (*n* = 4) and AD human cases (*n* = 4) as negative and positive controls. The lysates were immunoblotted for the presence and relative levels of sortilin, human APP, and p-tau protein products. The ~ 100 kDa sortilin band, representing the full-length protein, was blotted in all lysates, with the median levels comparable between the C57BL/6 mouse, transgenic, and human samples (*P* = 0.083, Kruskal-Wallis *H* test; *H* = 8.23) (Fig. 6a, c1). The ~ 15 kDa sortilin band was essentially not visible in immunoblots of the C57BL/6 and all the transgenic mouse lysates, but it was blotted in the human samples (Fig. 6a), with densitometry indicating a significant difference of the median between all mouse strains relative to the human cases (*P* = 0.021; Kruskal-Wallis *H* = 11.6) (Fig. 6c2). Full-length human APP as detected by the 6E10 antibody occurred in immunoblots of the transgenic and human lysates, with minimal signal seen in the C57BL/6 lysates (Fig. 6b). The protein levels were approaching a significant difference among the groups (*P* = 0.056; Kruskal-Wallis *H* = 14.6) but reached a significant difference between the C57BL/6 lysates relative to other groups (Fig. 6c3). Overall, the presence or relative amounts of human APP and p-tau products provided qualitative confirmation of the transgenic background of mouse tissues relative to negative (C57BL/6) and positive (human) controls. It should be mentioned that the monkey brain samples used in the present study were obtained from original investigations designated for neuroanatomical characterization only; therefore, no frozen tissues were available for biochemical analyses.

## Discussion

The scientific community has experienced great difficulty developing effective AD therapy despite decades’ worth of efforts. As failed drug trials are repeatedly announced, concerns arise as to whether the molecular drug target(s), the model system(s) used in preclinical tests, or the designs of clinical trials (e.g., beginning trials on predemented vs. demented patient cohorts) are sufficiently suited for successfully delivering mechanism-based medicine [12–17]. A further point is whether certain as yet unforeseen but functionally relevant pathologies of human AD remain to be integrated into preclinical drug-testing models to allow more predictable clinical trial outcomes. Thus, deepening the understanding of human AD pathology and pathogenesis, including exploration and validation of the inclusiveness of human-type neuropathology in animal models, remains not only important and necessary but also might extend novel cutting-edge to advance basic and translational research toward better disease diagnosis and care.

Many studies have been carried out to characterize the neuropathology in transgenic AD mouse models and aged nonhuman primates. In short, age-related cerebral amyloidosis involving parenchymal and vascular Aβ deposition has been well demonstrated in APP/PS1, 5×FAD, and 3×Tg-AD mice, as well as in aged rhesus and cynomolgus monkeys. Aβ deposition in these animal brains can arrange in the neuritic plaque type, such as that seen in the human brain [4–6, 8, 64, 65]. Apart from a similar pattern of Aβ deposition, plaque-associated dystrophic neurites exhibit similar neurochemical alterations. Specifically, accumulations of the amyloidogenic proteins (APP, BACE1, and PS1), axonal/presynaptic markers (e.g., synaptophysin, neurofilaments, and tubulins), neurotransmitter markers of multitype neurons (glutamatergic, GABAergic, cholinergic, catecholaminergic, and neuropeptidergic neurons), lysosomal proteins (e.g., LAMP1, cathepsins), and other proteins (e.g., some neurotrophic factors, reticulon 3) are observed in the dystrophic neurites in transgenic mouse, monkey, and human brains [42, 64, 65, 74–83]. Based on pattern comparison of Aβ and BACE1 labeling between adjacent sections and double-immunofluorescence assessment in the same sections, a large amount of parenchymal plaque in the cerebral cortex of aged rhesus and cynomolgus monkeys appears to be neuritic ([42] and the present study).

However, there is discussion of transgenic mouse models only partially recapitulating the pathological and clinical characteristics of human AD in respect to such as the extent of neuronal death, tau pathology (as seen only in the 3×Tg-AD mice), and inflammation, as elaborated in previous work [11–14, 84–86]. The present study extends strong evidence for a “qualitative” difference between humans and transgenic mice, and even monkeys, regarding the existence of extracellular sortilin pathology. Thus, neuritic plaques in humans are constituently different relative to rodents and nonhuman primates, because the plaques in the human brain additionally contain sortilin fragments. This also means that neuritic plaques in transgenic model mouse brains actually represent an incomplete form of this lesion as a pathological hallmark of AD. Therefore, it is reasonable to recommend that in future development of AD models, investigators should make an effort to add strategies that allow the formation of human-like extracellular sortilin pathology in the animal brain.

The present data support our initial assessment for a lack of an interdependent relationship between Aβ and sortilin deposition in the human brain, which was based on the preferential localization of sortilin deposits to neuritic plaques but not to typical diffuse Aβ plaques or amyloid vasculature [61]. In fact, because extracellular Aβ products and dystrophic neurites (as labeled with BACE1 antibody) are clearly present in the transgenic mouse as well as the aged monkey brain, the lack of extracellular sortilin pathology in these animals indicates that the sortilin deposition is not caused by passive Aβ absorption or by dystrophic axonal pathology. Our Western blot analysis results indicate that the ~ 15 kDa sortilin fragments are not readily detectable in the transgenic mouse cortical lysates, in line with the notion that they represent an important part of the extracellularly deposited sortilin product seen in the human brain. Together, the data derived our present and previous studies suggest that there exist species-related factor(s) governing the formation of sortilin fragments in the mammalian brain.

In this study we assessed p-tau in histological and immunoblot preparations mainly as an adjunctive measure for the purpose of assay control (sample verification). The two p-tau antibodies used show extensive labeling of tangled neuronal somata and processes in the human brain samples. In contrast, no neuronal profiles with enhanced p-tau reactivity are observed in aged monkey tissues. In the literature, findings of tauopathy in the squirrel and the rhesus and cynomolgus monkey brains have been inconsistent between reports [24, 35–39, 43]. Overall, it appears that macaques exhibit limited tau hyperphosphorylation and accumulation in neurons during brain aging [50]. Notably, during the preparation of this paper, researchers in a new study published data supporting a role for sortilin in the spread of tau prions in transgenic (tau-_P301S_) mice [62]. Thus, it would be of interest to explore if the lack of extracellular sortilin neuropathology also relates to the limited cerebral tauopathy in monkeys.

One appealing concept about AD is that this neurodegenerative disorder may be unique to humans [47–50]. *Homo sapiens* shares a great deal of genetic homogeneity (98–99%) with nonhuman primates, although huge differences exist between humans and other primates in anatomy, physiology, and behavior, with cognitive capability being the most remarkable watershed. It could be challenging to define the precise biological underpinning responsible for a “human-specific” AD susceptibility. Interspecies comparative pathological studies might provide some initial clues for further analysis. Related to this matter, it would be important to determine the presence or absence of extracellular sortilin pathology in macaques at more advanced ages, in other nonhuman primates, and in nonprimate animals. Such studies are important to clarify whether the observed sortilin neuropathology indeed represents a lesion unique to humans, thereby potentially contributing to human AD vulnerability. Alternatively, these studies could help identify pathologically more relevant natural animal models among existing candidates for basic and translational research in AD, including identification of the cellular and molecular factors involved in the formation of sortilin fragments, which are difficult to address using endpoint postmortem human brains.

## Conclusions

On the basis of lack of cerebral extracellular sortilin pathology in APP/PS1, 5×FAD and 3×Tg-AD mice, and aged macaques bearing overt cerebral β-amyloid deposition, we conclude that neuritic plaques of humans are constituently different relative to rodents and nonhuman primates. Specifically, neuritic amyloid plaques seen in transgenic mouse models of AD actually represent an incomplete form of this disease hallmark pathology. The human-specific extracellular sortilin pathology also implies a greater brain proteopathy in humans relative to rodents and nonhuman primates during aging and in AD.

## Additional file

Additional file 1: **Figure S1.** Sortilin immunolabeling with the C-terminal antibody in a frontal lobe section of an aged rhesus monkey at the level of the anterior end of the striatum (St), as indicated. **Figure S2.** β-Amyloid (Aβ) immunolabeling with the monoclonal 6E10 antibody in a frontal lobe section of an aged rhesus monkey at the level of the anterior end of the striatum (St), as indicated. **Figure S3.** β-Secretase (BACE1) immunolabeling with a well-characterized rabbit antibody in a frontal lobe section of an aged rhesus monkey at the level of the anterior end of the striatum (St). **Figure S4.** Pattern of labeling revealed with the PHF1 monoclonal phosphorylated tau antibody in a frontal lobe section of an aged rhesus monkey at the level of the anterior end of the striatum (St). **Figure S5.** Sortilin immunolabeling with the C-terminal antibody in a temporal lobe section of an aged cynomolgus monkey at the level passing the anterior hippocampus and the lateral geniculate nucleus (LGN). **Figure S6.** β-Amyloid (Aβ) immunolabeling with the monoclonal 6E10 antibody in a temporal lobe section of an aged cynomolgus monkey at the level of the anterior hippocampus. Extracellular amyloid plaques are present in a greater amount in the medial and lateral parts of the parietal neocortex (PC) and temporal neocortex (TC), relative to the entorhinal cortex (Ent). **Figure S7.** β-Secretase (BACE1) immunolabeling across the hemispheric section from an aged rhesus monkey passing the lateral geniculate nucleus (LGN) and anterior hippocampus. Neuropil-like reactivity is present over the cortical gray matter. **Figure S8.** Non-edge-montaged Motic microscopic image covering the area of the entire hemispherical section from an aged cynomolgus monkey at the level passing the anterior hippocampus and the lateral geniculate nucleus (LGN). (PDF 1960 kb)
