# Supplementary material for: Lack of human-like extracellular sortilin neuropathology in transgenic Alzheimer’s disease model mice and macaques
Source: Alzheimers Res Ther. 2018 Apr 24;10:40. doi: 10.1186/s13195-018-0370-2 (PMC5978992; doi:10.1186/s13195-018-0370-2)
Supplement: Supplementary file 1 — Figure S1. Sortilin immunolabeling with the C-terminal antibody in a frontal lobe section of an aged rhesus monkey at the level of the anterior end of the striatum (St), as indicated. Figure S2. β-Amyloid (Aβ) immunolabeling with the monoclonal 6E10 antibody in a frontal lobe section of an aged rhesus monkey at the level of the anterior end of the striatum (St), as indicated. Figure S3. β-Secretase (BACE1) immunolabeling with a well-characterized rabbit antibody in a frontal lobe section of an aged rhesus monkey at the level of the anterior end of the striatum (St). Figure S4. Pattern of labeling revealed with the PHF1 monoclonal phosphorylated tau antibody in a frontal lobe section of an aged rhesus monkey at the level of the anterior end of the striatum (St). Figure S5. Sortilin immunolabeling with the C-terminal antibody in a temporal lobe section of an aged cynomolgus monkey at the level passing the anterior hippocampus and the lateral geniculate nucleus (LGN). Figure S6. β-Amyloid (Aβ) immunolabeling with the monoclonal 6E10 antibody in a temporal lobe section of an aged cynomolgus monkey at the level of the anterior hippocampus. Extracellular amyloid plaques are present in a greater amount in the medial and lateral parts of the parietal neocortex (PC) and temporal neocortex (TC), relative to the entorhinal cortex (Ent). Figure S7. β-Secretase (BACE1) immunolabeling across the hemispheric section from an aged rhesus monkey passing the lateral geniculate nucleus (LGN) and anterior hippocampus. Neuropil-like reactivity is present over the cortical gray matter. Figure S8. Non-edge-montaged Motic microscopic image covering the area of the entire hemispherical section from an aged cynomolgus monkey at the level passing the anterior hippocampus and the lateral geniculate nucleus (LGN). (PDF 1960 kb) [file 13195_2018_370_MOESM1_ESM.pdf]

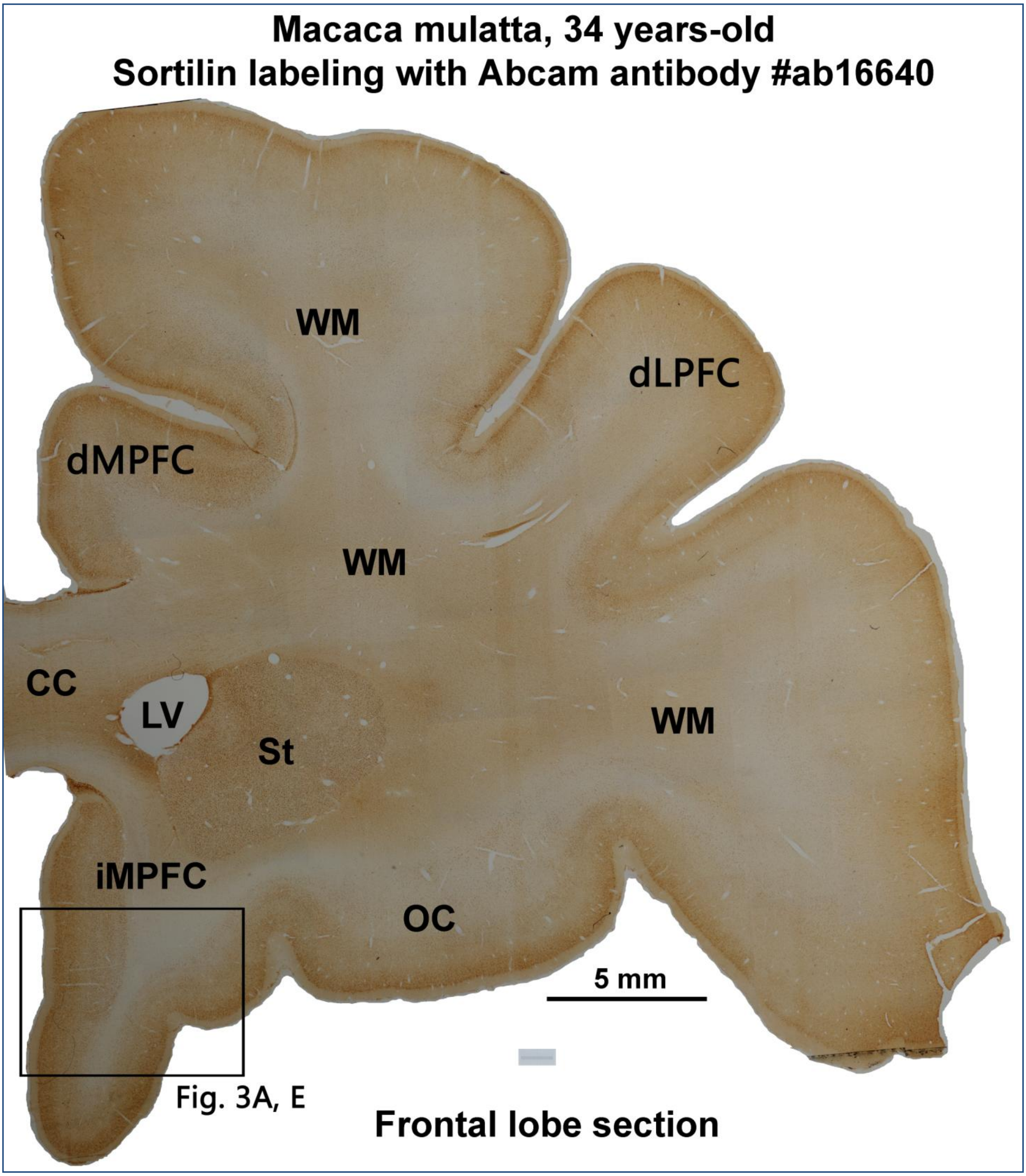

Figure S1: Sortilin immunolabeling with the C-terminal antibody in a frontal lobe section of an aged rhesus monkey at the level of the anterior end of striatum (St), as indicated. Note that there is no plaque-like extracellular labeling across the entire section. Also see labeling for β-amyloid, BACE1 and p-Tau in the sections at the same frontal level in Supplemental Figures 2-4. WM: white matter; iMPFC: inferior medical prefrontal cortex; dMPFC: dorsal medical prefrontal cortex; dLPFC: dorsal lateral prefrontal cortex; CC: corpus callosum; LV: lateral ventricle; OC: orbit cortex. The framed area is shown as Figure 3A, E in the paper. Scale bar = 5mm.

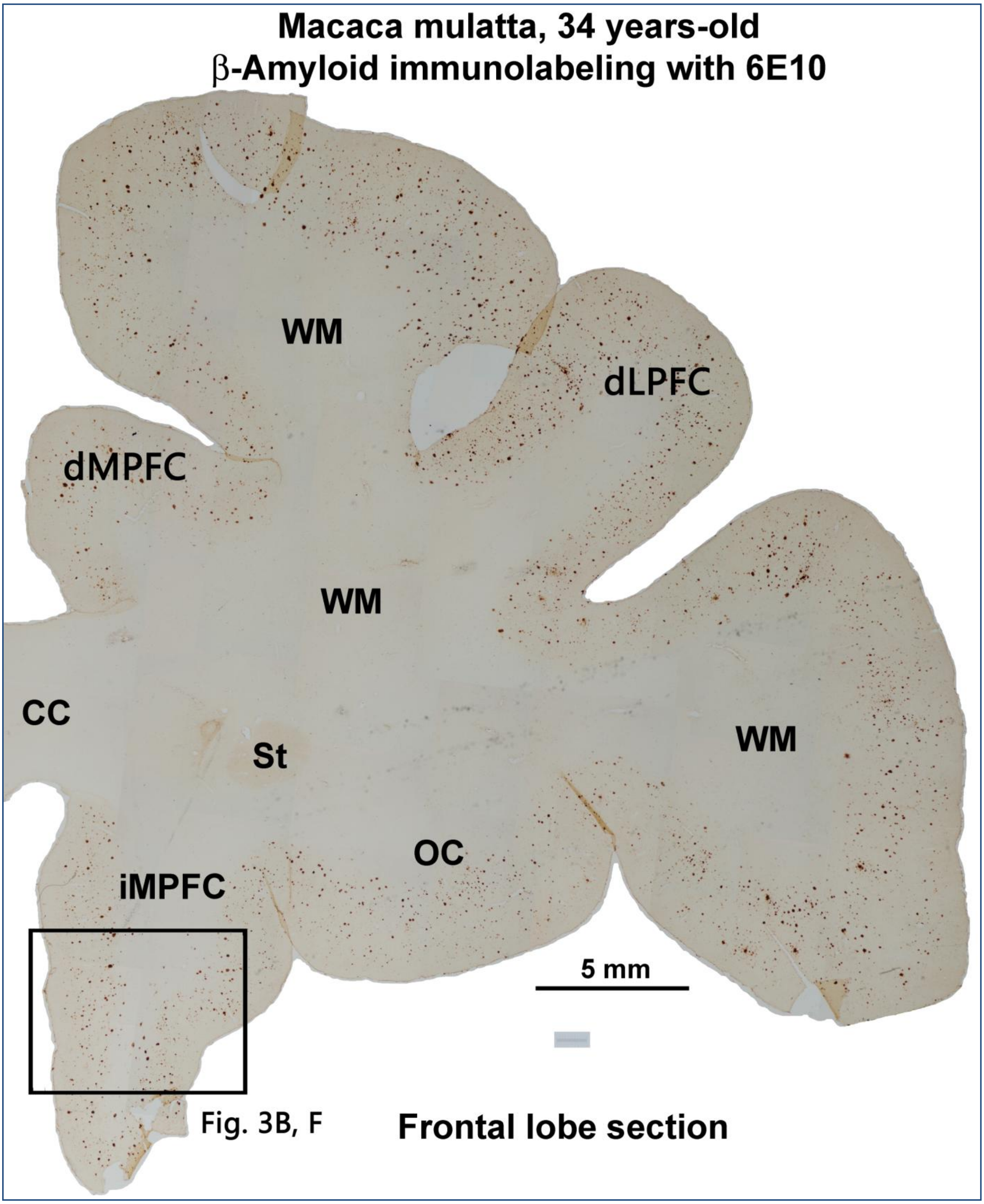

Figure S2: β-Amyloid (Aβ) immunolabeling with the monoclonal 6E10 antibody in a frontal lobe section of an aged rhesus monkey at the level of the anterior end of striatum (St), as indicated. Note the wide spread extracellular plaque labeling across the entire section. The framed area is shown as Figure 3B, F in the paper. Abbreviations are as defined in Supplemental Figure 1. Scale bar = 5mm.

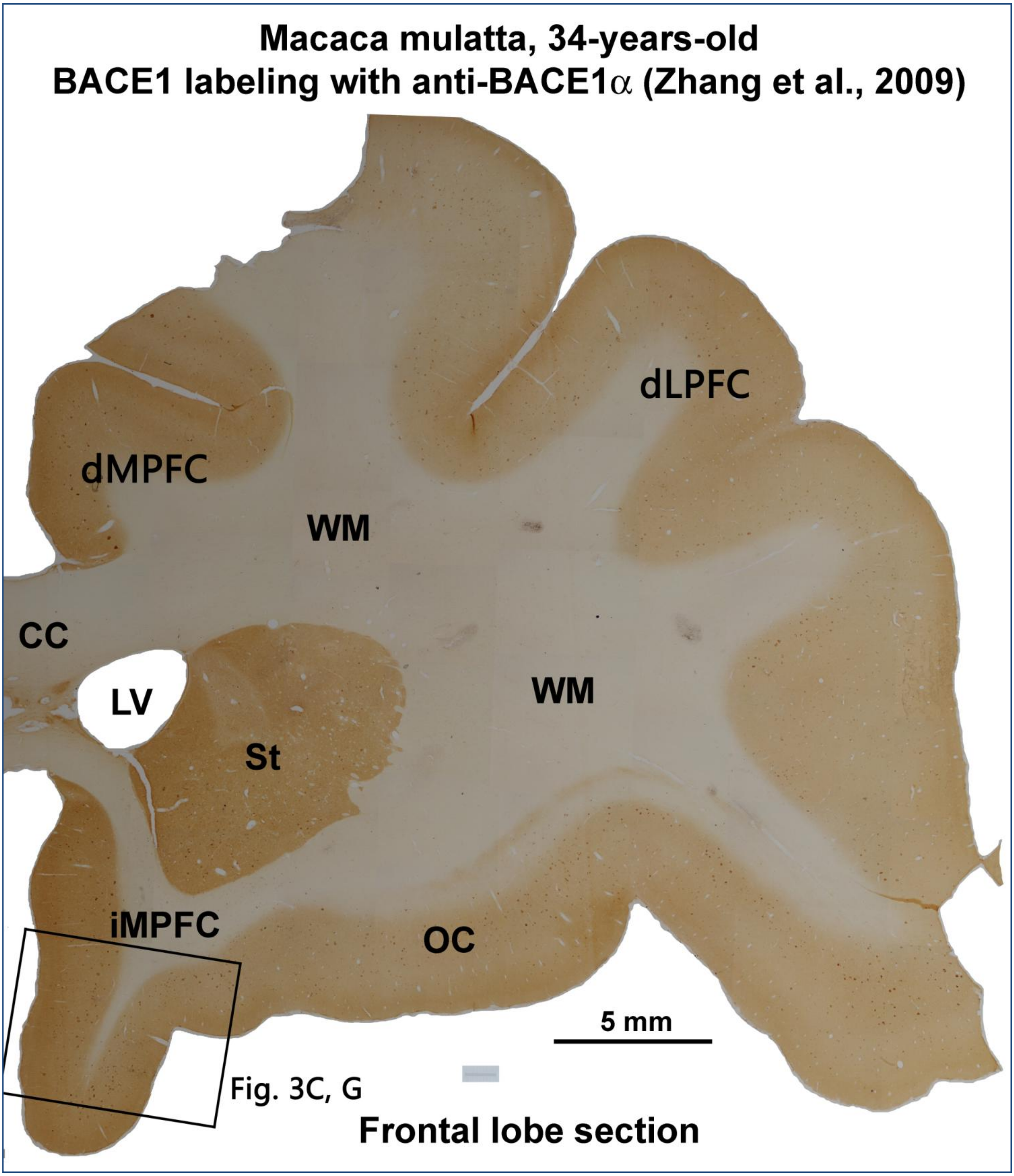

Figure S3: β-Secretase (BACE1) immunolabeling with a well characterized rabbit antibody in a frontal lobe section of an aged rhesus monkey at the level of the anterior end of striatum (St). The labeling exhibits a neuropil-like pattern over the cortex. By enlarging the image on the screen, clusters of dystrophic neurites are seen to show increased reactivity relative to background and they distribute over the cortex across the section. The framed area is shown as Figure 3C, G in the paper. Abbreviations are as defined in Supplemental Figure 1. Scale bar = 5mm.

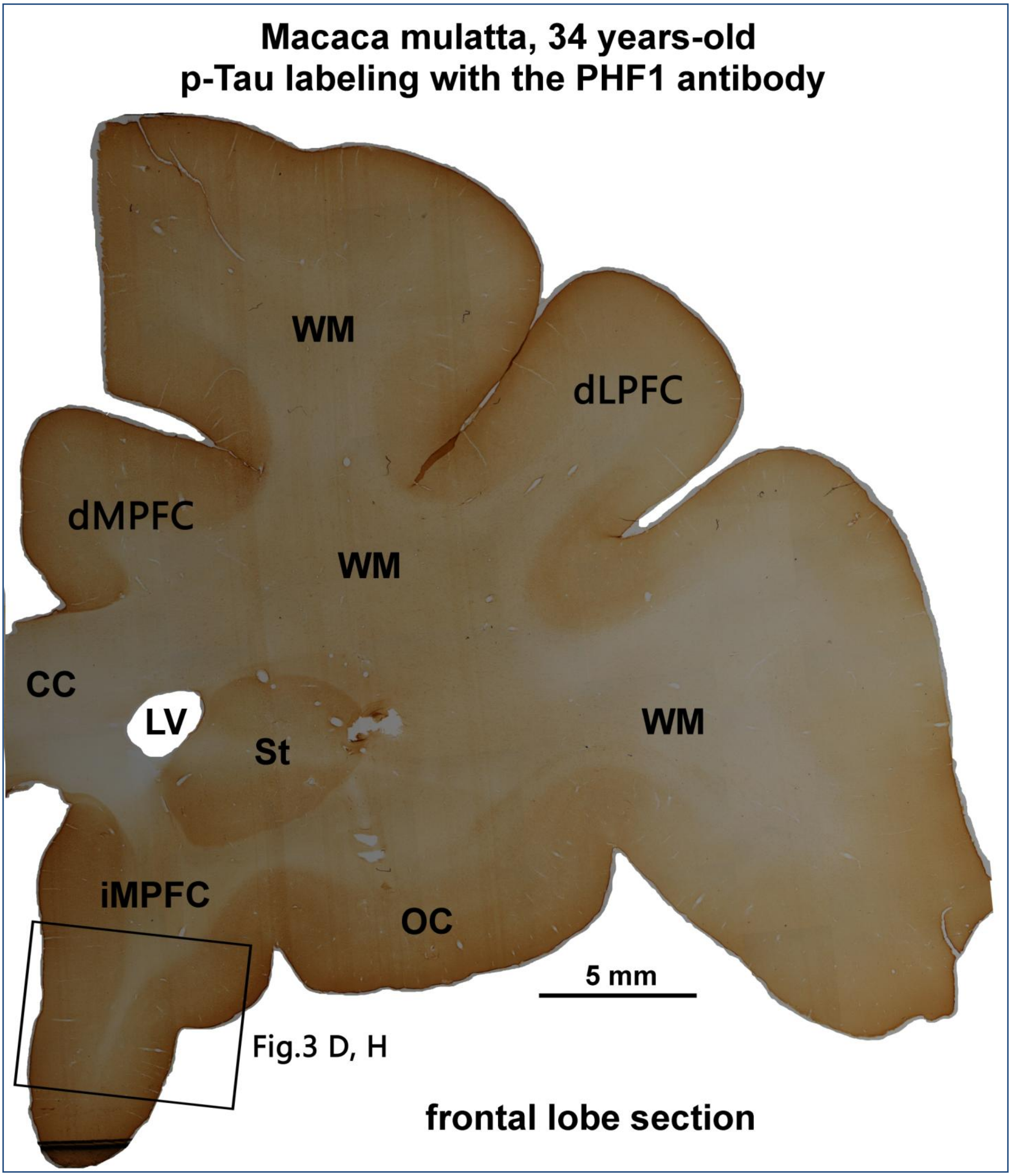

Figure S4: Pattern of labeling revealed with the PHF1 monoclonal phosphorylated tau antibody in a frontal lobe section of an aged rhesus monkey at the level of the anterior end of striatum (St). The labeling is background-like without any cellular profiles identifiable across the entire area of the section. The framed area is shown as Figure 3D, H in the paper. Abbreviations are as defined in Supplemental Figure 1. Scale bar = 5mm.

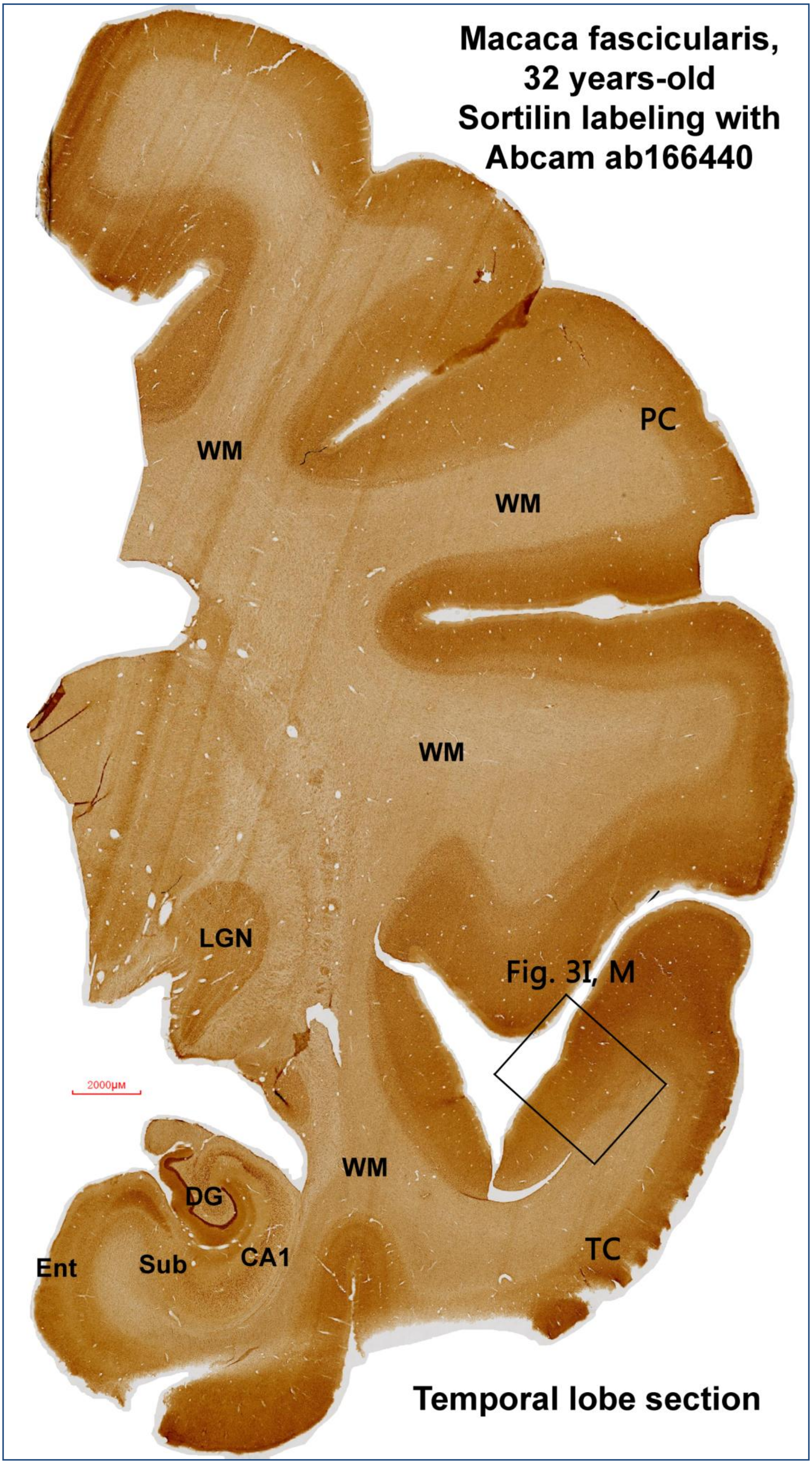

Figure S5: Sortilin immunolabeling with the C-terminal antibody in a temporal lobe section of an aged cynomolgus monkey at the level passing the anterior hippocampus and the lateral geniculate nucleus (LGN). As with the pattern seen in Supplemental Figure 1, there is no plaque-like extracellular labeling across the entire section. Note the strong labeling in the granule cell and molecular layer of the dentate gyrus (DG). Labeling for β-amyloid, BACE1 and p-Tau in adjacent the sections is shown as Supplemental Figures 6-8. WM: white matter; PC: parietal cortex; TC: temporal cortex; Ent: entorhinal cortex; Sub: subiculum; LV: lateral ventricle; OC: orbit cortex. The framed area is shown as Figure 3I and M in the paper. Scale bar = 2 mm.

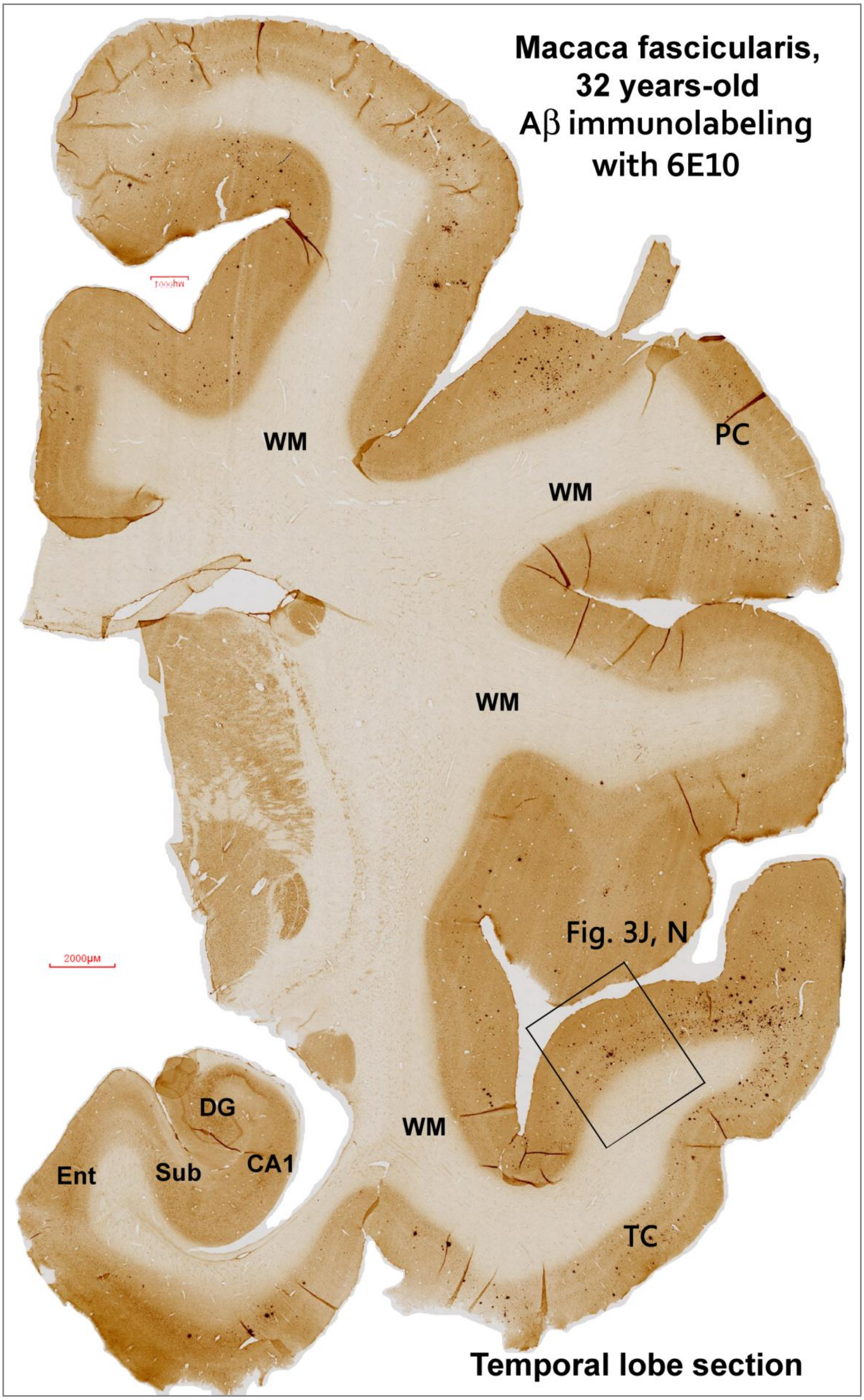

Figure S6: β-Amyloid (Aβ) immunolabeling with the monoclonal 6E10 antibody in a temporal lobe section of the aged cynomolgus monkey at the level of the anterior hippocampus. Extracellular amyloid plaques are present in a greater amount in the medial and lateral parts of the parietal neocortex (PC) and temporal neocortex (TC), relative to the entorhinal cortex (Ent). Very few plaques are seen in the hippocampal formation. The framed area with fairly dense plaques is shown as Figure 3J, N in the paper. Abbreviations are as defined in Supplemental Figure 5. Scale bar = 2 mm.

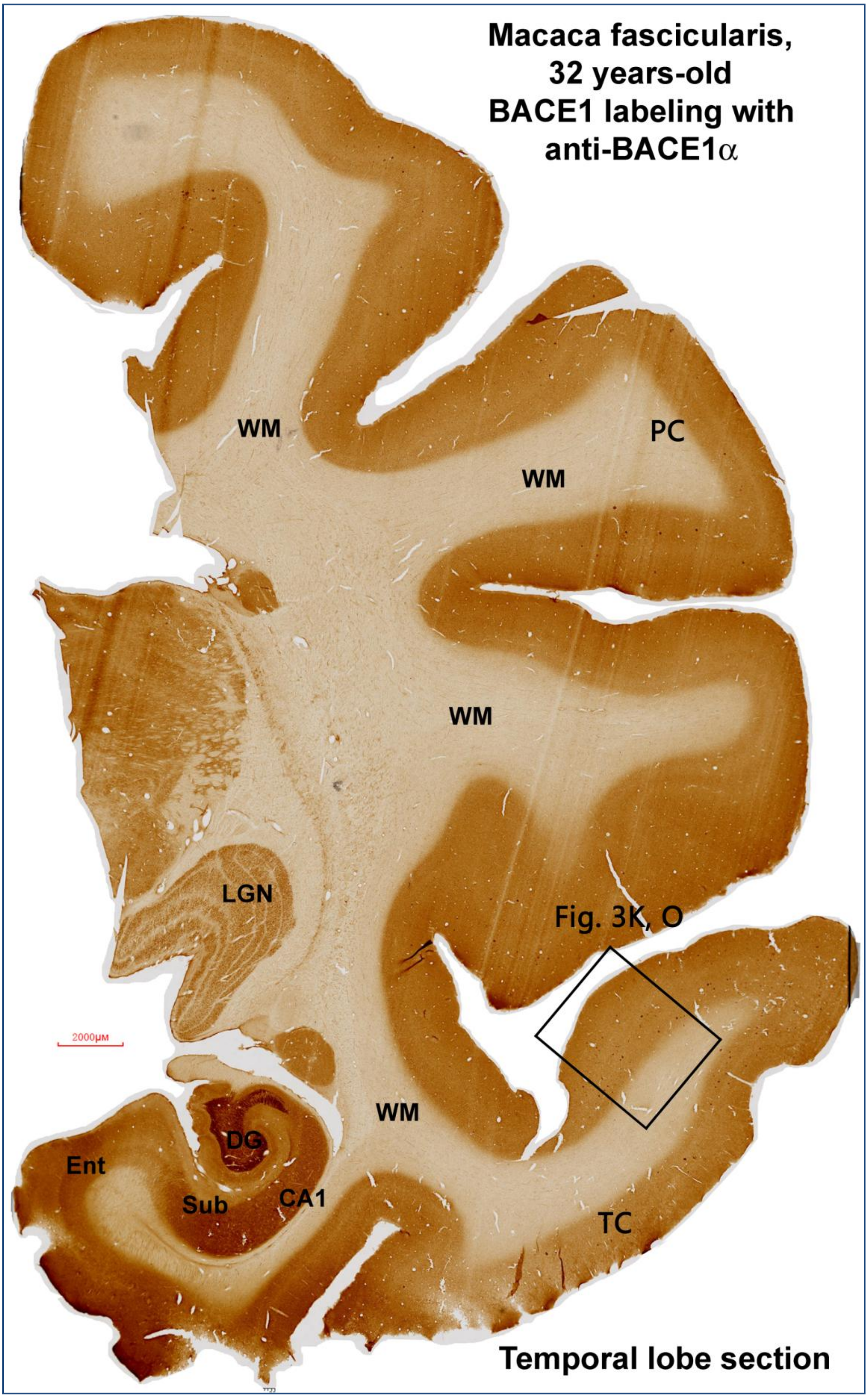

Figure S7: β-Secretase (BACE1) immunolabeling across the hemispheric section from the aged rhesus monkey passing the lateral geniculate nucleus (LGN) and anterior hippocampus. Neuropil-like reactivity is present over the cortical gray matter. Heavy labeling is present in the dentate gyrus (DG) in association with mossy fiber terminals. There are clusters of dystrophic neurites with increased reactivity relative to background, visible by enlarging the image. These neuritic profiles also appear more frequent in upper parts of the parietal neocortex and superior temporal neocortex. The framed temporal cortical area is shown as Figure 3K and O in the paper. Abbreviations are as defined in Supplementary Figure 5. Scale bar = 2 mm.

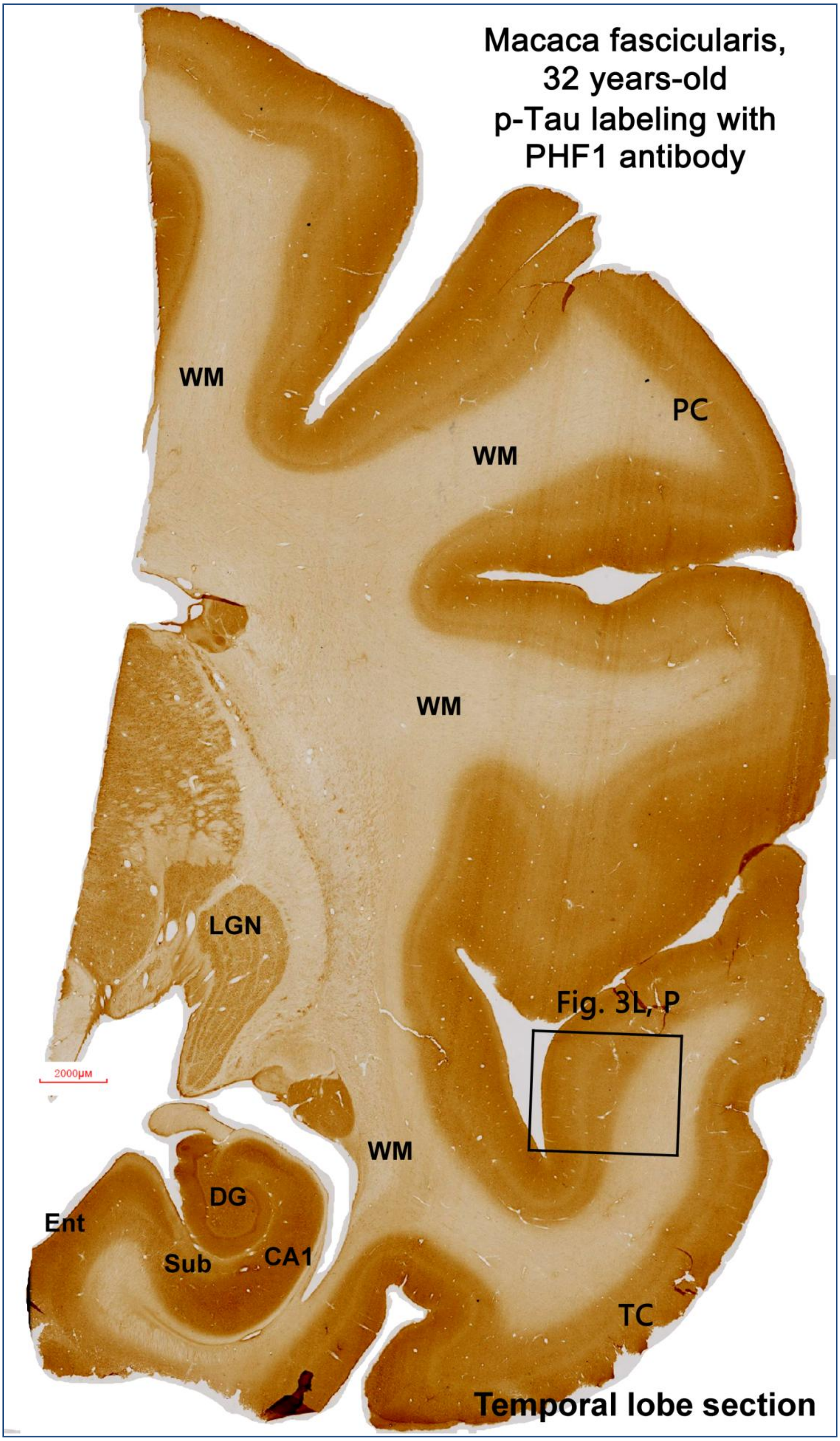

Figure S8: Non-edge montaged Motic-microscopic image covering the area of the entire hemispherical section from an aged cynomolgus monkey at the level passing the anterior hippocampus and the lateral geniculate nucleus (LGN). This figure illustrates the immunolabeling pattern revealed with the PHF1 monoclonal phosphorylated tau antibody. No cellular profiles resembling neurofibrillary tangle lesions seen in Alzheimer's disease human brain are detectable over the cerebral cortex or the hippocampal formation. The framed area of the temporal neocortex is enlarged and shown as Figure 3L, P in the paper. Abbreviations are as defined in Supplemental Figure 5. Scale bar = 2 mm.
